# Strategies in anti-*Mycobacterium tuberculosis* drug discovery based on phenotypic screening

**DOI:** 10.1038/s41429-019-0205-9

**Published:** 2019-07-11

**Authors:** Edyta M. Grzelak, Mary P. Choules, Wei Gao, Geping Cai, Baojie Wan, Yuehong Wang, James B. McAlpine, Jinhua Cheng, Yingyu Jin, Hanki Lee, Joo-Won Suh, Guido F. Pauli, Scott G. Franzblau, Birgit U. Jaki, Sanghyun Cho

**Affiliations:** 1 Institute for Tuberculosis Research, College of Pharmacy, University of Illinois at Chicago, Chicago, IL USA; 2 Department of Medicinal Chemistry & Pharmacognosy, College of Pharmacy, University of Illinois at Chicago, Chicago, IL USA; 3 Center for Nutraceutical and Pharmaceutical Materials, Myongji University, Cheoin-gu, Gyeonggi-do Republic of Korea; 4 Division of Bioscience and Bioinformatics, College of Natural Science, Myongji University, Cheoin-gu, Gyeonggi-do Republic of Korea; 5 Interdisciplinary Program of Biomodulation, Myongji University, Cheoin-gu, Gyeonggi-do Republic of Korea

**Keywords:** Phenotypic screening, Drug discovery and development

## Abstract

The rise of multi- and extensively drug-resistant *Mycobacterium tuberculosis* (*M. tb*) strains and co-infection with human immunodeficiency virus has escalated the need for new anti-*M. tb* drugs. Numerous challenges associated with the *M. tb*, in particular slow growth and pathogenicity level 3, discouraged use of this organism in past primary screening efforts. From current knowledge of the physiology and drug susceptibility of mycobacteria in general and *M. tb* specifically, it can be assumed that many potentially useful drug leads were missed by failing to screen directly against this pathogen. This review discusses recent high-throughput phenotypic screening strategies for anti-*M. tb* drug discovery. Emphasis is placed on prioritization of hits, including their extensive biological and chemical profiling, as well as the development status of promising drug candidates discovered with phenotypic screening.

## Introduction

Tuberculosis (TB) is among the ten leading causes of death worldwide and the leading cause from a single infectious bacterium [1]. In 2017, 10 million people developed TB disease, 558,000 of which were resistant to rifampicin, the most effective first-line drug [1]. The spread of drug-resistant TB and the concomitant interaction with the human immunodeficiency virus (HIV) epidemic, increases the challenges associated with TB control and treatment [2, 3]. New diagnostic tools, more effective drugs and adjunct therapies are urgently needed to improve the treatment outcome [4].

The rate of progress in anti-*Mycobacterium tuberculosis* (*M. tb*) drug discovery is limited by many technical challenges. *M. tb* is a slow growing, most commonly respiratory, pathogen that requires handling in Biosafety Level 3 (BSL3) facilities. The lipid-rich nature of the mycobacterial cell wall contributes to its low permeability and prevents many small molecules from accessing internal molecular targets. Active multi-drug efflux pumps account for one of the many mechanisms of resistance to antibiotics [5]. Within the host, *M. tb* exists as a heterogeneous population in different microenvironments, which requires drugs to be active against multiple physiological states under various conditions [6]. Furthermore, the ideal new anti-*M. tb* drug should be safe and show minimal drug–drug interactions with other antimicrobials used for treatments or major side effects over an extended treatment period of six months to 2 years. Ideally, the new regimens would decrease required treatment durations leading to increased patient compliance and improved outcomes. New leads should also be active against drug-resistant *M. tb* strains and be compatible with HIV therapy [7].

Historically, many antibiotic lead compounds were isolated from soil-derived actinomycetes during the golden era of antibiotic discovery in the mid-20th century. The original discovery platform consisted of screening for antimicrobial activity against susceptible bacteria using agar-based diffusion or cross-streak methods. These simple methods also led to the discovery of the first anti-*M. tb* drug, streptomycin [8], and provided a starting point for the discovery of the major classes of antibiotics [9]. Several decades later, the repeated isolation of known compounds and the emergence of antibiotic-resistance shifted the focus to semi-synthetics to overcome resistance and broaden the activity spectrum. Combinatorial chemistry fueled the development of synthetic antimicrobials and high-tech platforms for screening of large compound libraries. Addressing this demand, high-throughput screening (HTS) was introduced as part of industrial drug discovery platforms [10].

## Selection of strain and type of HTS assay

The bacteria used in phenotypic (whole-cell) screens of most of the early and some of the current anti-*M. tb* drug discovery efforts were principally *M. bovis* (BCG) or *M. smegmatis*, to circumvent the issues of slow growth and/or safety, while *M. tb* was used to a much lesser extent. Considering the abundant use of surrogate pathogens in anti-*M. tb* screening, it is reasonable to hypothesize that many potentially useful *M. tb* drug leads have been missed in the past.

The parallel screens of ~5000 compounds from the Library of Pharmacologically Active Compounds (LOPAC), NIH Diversity Set and Spectrum Collection were performed to evaluate the efficacy of *M. smegmatis* and *M. bovis* as surrogate in vitro models [11]. The data analysis revealed that 48–50% of the *M. tb* inhibitors could not be detected when screening against *M. smegmatis*, and 21% were missed in *M. bovis* screens. The genomic comparisons indicated that 30% of *M. tb* proteins lack conserved orthologues in *M. smegmatis* and 3% in *M. bovis*. Although, *M. bovis* represents a more sensitive model, *M. smegmatis* is often favored in HTS due to its faster growth, 4 days, compared with 14 days for *M. bovis*. Bedaquiline (TMC207), the first FDA approved TB drug for 40 years, was identified by HTS with *M. smegmatis* as surrogate [12].

Phenotypic screening opens the path to discovery of compounds that inhibit new target(s) or pathway(s). However, this approach does not provide information about the drug target. The determination of the whole *M. tb* genome sequence in 1998 led to the identification of potential drug targets and development of new target-based screens [13]. Nevertheless, the target-based HTS approach does not necessarily translate cell-free assay activity to whole-cell activity. There have been only a few targets that were validated, and for many essential ones there are no specific inhibitors with drug-like properties. The target-based approach has yet to yield a clinically useful anti-mycobacterial agent [14].

Manjunatha and Smith reported experiences gained from TB drug discovery at the Novartis Institute for Tropical Disease (NITD) in Singapore [15]. Both, molecular target and phenotypic screenings were pursued to identify new compounds active against multi-drug-resistant TB (MDR-TB) and extensively drug-resistant TB (XDR-TB). The lack of success with the target-based strategy shifted the efforts to phenotypic screens. The primary screening against *M. bovis* BCG with ~2.2 million Novartis compounds was followed by hit confirmation against *M. tb* H37Rv. The work from hit to lead took over 7 years and five chemical series—pyrimidineimidazoles, imidazopyridines, indolcarboxamides, pyrazolopyrimidines, and pyridones [15].

## Promising anti-*M. tb* drug candidates from HTS

**Fig. 1:**
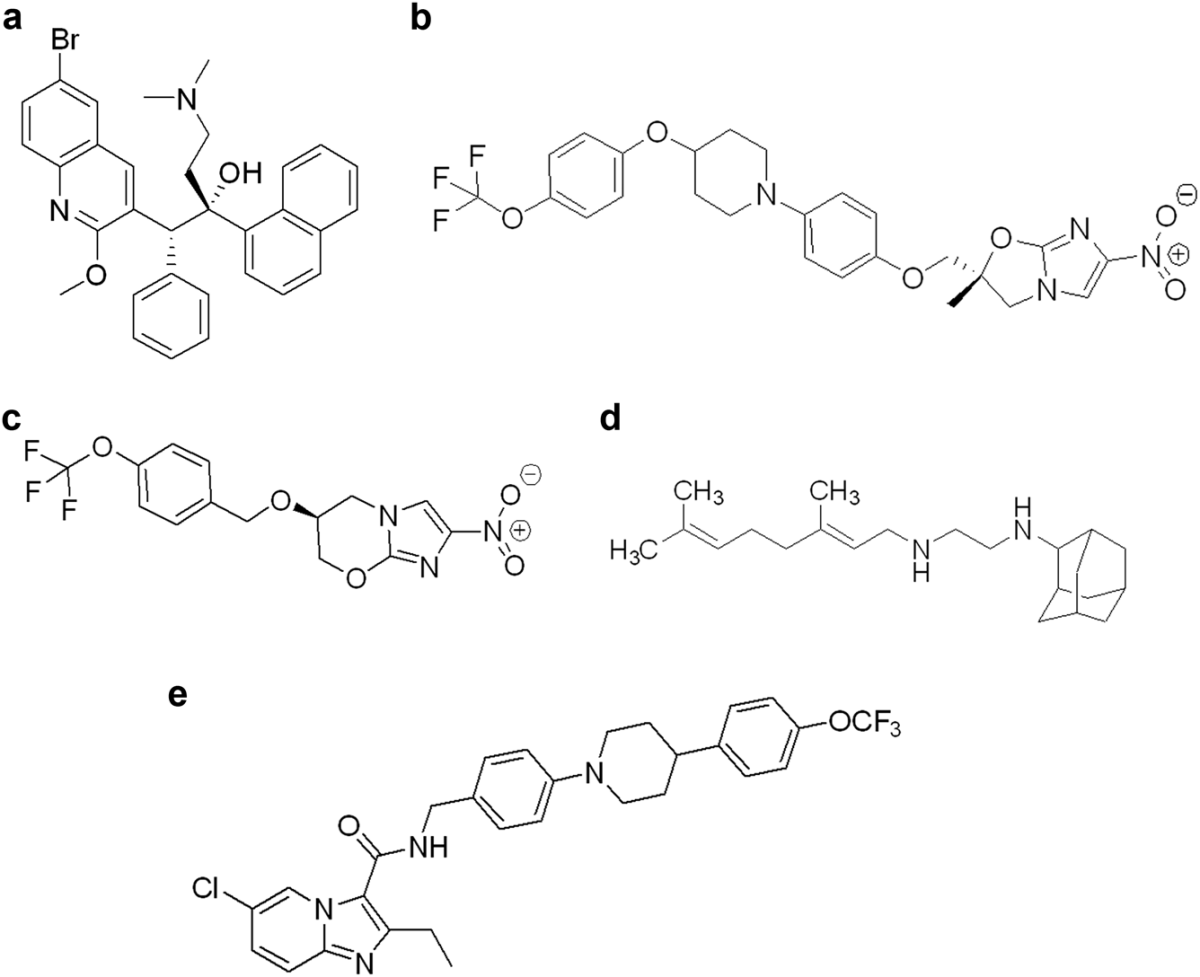
The chemical structures of anti-*M. tb* compounds in clinical trials: **a** bedaquiline, **b** delamanid, **c** pretomanid, **d** SQ109, and **e** Q203

Notably, all recently introduced anti-*M. tb* drugs and promising candidates in clinical trials resulted from drug-to-target paths that involved HTS against whole cells (Fig. 1).

As previously noted, bedaquiline (TMC207), a diarylquinoline, was identified in a screen against *M. smegmatis* by Janssen Pharmaceuticals [12]. It is highly active against *M. tb* including MDR- and XDR-TB. It also shows in vitro and in vivo activity against non-tuberculous mycobacteria including *M. avium* complex, *M. abscessus*, and *M. kansasii*. Bedaquiline has a novel target and mechanism of action; it inhibits the function of adenosine 5′-triphosphate (ATP) synthase and the subsequent energy supply [16]. In 2012, bedaquiline was approved by the FDA for the treatment of pulmonary MDR-TB as part of an appropriate combination therapy in adult patients with resistance or intolerability to other treatment regimens. However, due to limited data and concerns related to higher death rates among patients who received bedaquiline in the phase II randomized controlled trial, the WHO advises caution when administering this medication [17]. Numerous ongoing phase II and III clinical trials are evaluating efficacy and safety of bedaquiline in HIV-infected and HIV-uninfected participants with MDR-TB. The recent outcomes from five cohorts of patients with MDR-TB treated with bedaquiline for >6 months as a part of standard background regimens reported 78% culture conversion to negative at 6 months, 65.8% treatment success rate and 11.7% death rate. These results compare favorably with those observed in large cohorts of patients with MDR-TB in the pre-bedaquiline era with success rates of 54–58% and death rates of 13.8–15% [18].

The nitroimidazoles, delamanid (OPC-67683), and pretomanid (PA-824), are analogs of azomycin, originally isolated from *Streptomyces eurocidicus* [19]. They are highly active against replicating and non-replicating *M. tb*, which brings the potential to shorten duration of the treatment. Delamanid and pretomanid appear to have a multi-target mechanism of action; acting on the inhibition of cell wall biosynthesis through inhibition of methoxy- and keto-mycolic acid synthesis and respiratory poisoning through release of nitric oxide during bacterial drug metabolism [20–22]. Both compounds are prodrugs and are reductively activated by a deazaflavin (F420)-dependent nitroreductase (Ddn). Des-nitroimidazole products of Ddn generate reactive nitrogen species, including nitric oxide (NO) which promotes the anti-mycobacterial activity under anaerobic conditions [23–25]. Delamanid was developed by Otsuka Pharmaceutical and was approved for the treatment of MDR-TB in Japan and by the European Medicines Agency (EMA) in 2014 [26]. It has also been a promising candidate for the treatment of TB/HIV co-infections. The ongoing clinical trials evaluate, e.g., efficacy of a 9–12 month long treatment regimen, including delamanid, linezolid, levofloxacin, and pyrazinamide, for the treatment of quinolone-sensitive MDR-TB, and the pharmacokinetics, safety and tolerability of delmanid in combination with an optimized multi-drug background regimen (OBR) for MDR-TB in HIV-infected and HIV-uninfected children with MDR-TB. Currently, the WHO recommends to include delamanid in longer MDR-TB regimens only when patients cannot tolerate or show resistance to certain second-line drugs [27]. Pretomanid, was discovered by Pathogenesis Corporation and developed by the Global Alliance for TB Drug Development. Recently, a new drug application (NDA) for pretomanid has been accepted for review by the FDA and proposed the use of pretomanid as part of a new regimen, in combination with bedaquiline and linezolid, for the treatment of XDR- and MDR-TB (https://www.tballiance.org/news/pretomanid-enters-FDA-review).

The attempt to develop a new second-generation drug from the first-line drug ethambutol lead to the discovery of SQ109 [28, 29]. A library of 63,238 compounds based on a 1,2-ethylenediamine pharmacophore of ethambutol was synthesized and screened against *M. tb*. Among 69 hits, SQ109 was the most potent, and retained activity against isoniazid- and rifampicin-resistant *M. tb* strains. Activity against an ethambutol-resistant *M. tb* strain suggested a target, mode of action and/or activation pathways different than those of ethambutol. The SQ109 triple mode of action includes (i) the inhibition of MmpL3, a membrane transporter for trehalose monomycolate which is involved in cell wall synthesis of *M. tb*, (ii) the inhibition of the enzymes MenA and MenG, which are involved in menaquinone biosynthesis, and (iii) the reduction of ATP synthesis acting as an uncoupler [30]. SQ109 enhances activity of isoniazid, rifampicin, and bedaquiline and shortens the time required to cure mice of experimental TB by >30%. SQ109 was discovered by scientists at Sequella, Inc. (Rockville, MD), and the US National Institutes of Health. In 2011, Infectex, Ltd, licensed the rights to develop and commercialize SQ109 in the Russian Federation and Commonwealth of Independent States. In 2017, Infectex, Ltd in collaboration with Sequella, announced the results of a phase IIb-III clinical trial to assess efficacy, safety and tolerability of SQ109 in combination with a standard regimen for MDR-TB treatment. Patients treated with SQ109-containing regimens showed statistically significant improvement in clearance of *M. tuberculosis* in the lung.

A phenotypic HTS at the Pasteur Institute in Korea of a library of 121,156 different chemical compounds for their ability to inhibit *M. tb* growth in mouse macrophages lead to two series of imidazopyridine amides (IPA) [31]. They were found to be active in low micromolar range against bacteria replicating inside macrophages and in axenic culture medium. The synthesis and evaluation of 477 derivatives of the hit compound led to the optimized IPA, Q203 [31, 32]. The primary target of Q203 is the cytochrome unit bc_1_ complex which is an essential component of the electron transport chain required for ATP synthesis. The effect on energy metabolism and the potency at low doses in the chronic mouse model of TB suggest that Q203 may contribute to shortening the treatment time of TB. Q203 also appears to be synergetic with bedaquiline in the murine chronic infection model, indicating potential for new drug regimen. A phase II clinical trial by Qurient Co. Ltd is currently evaluating bactericidal activity, safety, tolerability, and pharmacokinetics of multiple oral doses of telacebec (Q203).

## Anti-*M. tb* drug discovery at the Institute for Tuberculosis Research (ITR)

Natural products still appear to be a promising source of new antibiotics. Approximately, 78% of the therapeutics in the area of infectious diseases derive from natural products. In this review, we share our experience from over a decade of effort that was made in the laboratories of the Institute for Tuberculosis Research (ITR) at the University of Illinois at Chicago, in collaboration with Myongji University in Republic of Korea, to discover new anti-*M. tb* drugs from soil-derived actinomycetes. Emphasis is placed on the workflow for HTS phenotypic screening against virulent *M. tb* and on hit prioritization using assays established in the ITR laboratories. The strategies and new approaches for isolation and identification of active leads is discussed as well. An anti-*M. tb* drug discovery platform is presented by means of the discovery of a group of anti-mycobacterial cyclic peptides that target ClpC1 in *M. tb* [33–36].

### Construction of the actinomycete extracts library and primary HTS screening

**Fig. 2:**
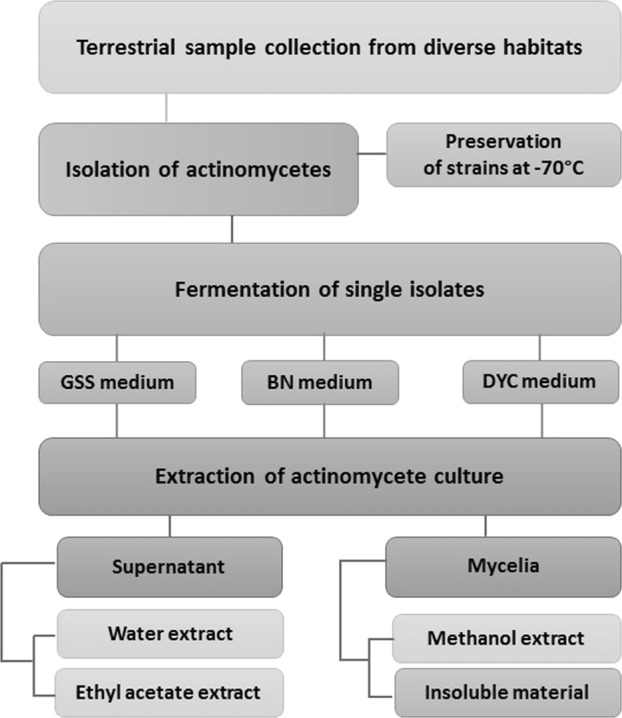
The scheme of preparation of the actinomycetes library

During the past decade in the ITR laboratories, over 200,000 actinomycete-derived extracts were screened directly against virulent *M. tb* H37Rv using a luxABCDE reporter and the microplate alamar blue assay (MABA). This unique library, the Extract Collection of Useful Microorganisms (ECUM) (www.ecum.or.kr), was established in 1993 at Myongji University in the Republic of Korea. It includes extracts of soil microorganisms collected from sites with distinctive ecologies and unusual weather conditions, such as polar, alpine, tropical, volcanic, and desert areas. Selected actinomycete isolates were cultured in three different liquid media, i.e., glucose-soybean starch (GSS), Bennett’s (BN), and dextrin-yeast-corn steep liquor (DYC). The cell pellet was then extracted with methanol, and the supernatant with ethyl acetate. As a result, nine different extracts were generated for each actinomycete isolate (Fig. 2; Supplementary data).

### HTS of actinomycete library against *M. tb*

The primary HTS of the ECUM library was performed with a whole-cell-based assay for growth inhibition of the luciferase expressing strain of *M. tb* H37Rv lux ABCDE in a 384-well format [37, 38] (Supplementary data). This type of assay was chosen to concurrently explore multiple targets of *M. tb* and remain open to finding compounds with a new mode of action. Assay quality and data variability were assessed by calculating the standard deviation (SD), screening window coefficient (Z-factor), signal-to-noise, and signal-to-background ratios [39]. A meticulous validation process was employed in order to address potential concerns about false positive and negative results (Fig. 2).

A total of seven HTS campaigns were conducted, collectively screening ~200,000 actinomycete extracts against replicating *M. tb* lux ABCDE. The results of the single-dose primary screen were expressed as percent inhibition, calculated according to Eq. (1).

1 $${\% }\,{\mathrm{Inhibition}} = \frac{{\rm{Mean}}_{\rm{bacteria}} - {\rm{Test}} }{{\rm{Mean}}_{\rm{bacteria}} -{\rm{Mean}}_{\rm{medium}}} \times 100{\mathrm{\% }}$$

All 2042 extract samples that exhibited ≥90% inhibition of luminescence relative to the untreated control cultures were designated as hits and re-tested against *M. tb* H37Rv.

Most of the active samples were found to be among the ethyl acetate (46%) and methanol (34%) extracts obtained from GSS (44%) and BN (35%) media. Only 20% of the hits originated from water extracts. The lower hit rate for the water extracts could be explained by the fact that larger hydrophilic compounds cannot penetrate the highly hydrophobic outer layer of *M. tb*. It was observed that only small hydrophilic compounds are able to permeate the *M. tb* cell wall via the same hydrophilic channels that enable access to polar nutrients [40].

### Confirmation of anti*-M. tb* activity and preliminary evaluation of safety profile

The confirmatory screening of the hits selected after the primary HTS included two previously established in vitro assays [38] (Supplementary data). The first assay determines the inhibition of actively growing *M. tb* under aerobic conditions, whereas the second one tests the compounds’ activity against non-replicating *M. tb* under hypoxic conditions. Primary hit identification was based on the assay results against replicating *M. tb*. Extracts with additional activity against non-replicating *M. tb* were prioritized due to the potential to shorten duration of TB treatment.

In addition to considering the activity against replicating and non-replicating *M. tb*, the identification of hits was also based on mammalian cell toxicity measurements. Primary cytotoxicity was evaluated against a green monkey kidney cell (Vero) line, and all samples with growth inhibition > 50% were deprioritized.

The subsequent prioritization step considered both the anti-*M. tb* MIC values and the IC_50_ vs Vero cells. The MABA determines the MICs of the extracts against replicating *M. tb* under aerobic conditions, while the low oxygen recovery assay (LORA) determines their MICs against non-replicating *M. tb* under hypoxic conditions. All calculations were based on the assumption that the concentration of the DMSO-reconstituted extracts was 1 mg/mL, and the MIC and IC_50_ values were then used to calculate the selectivity index (SI) as IC_50_/MIC against both replicating and non-replicating *M. tb*. The SI can be considered as a preliminary estimation of the extract’s therapeutic window. To prioritize highly promising extracts, a SI > 50 was used as a threshold.

### Biological dereplication

The risk of re-isolation of known compounds from natural products is significant. To focus efforts on new molecules/targets as much as possible, biological dereplication was introduced into the workflow by testing the hits against a panel of *M. tb* isolates with resistance to known antibiotics. This included mono-resistant strains to isoniazid (INH), rifampin (RIF), streptomycin (SM), kanamycin (KM), cycloserine (CS), bedaquiline (TMC207), and the in-house generated *M. tb* strains with mono-resistance to clofazimine (CLF), moxifloxacin (MOX), and capreomycin (CAP) (Supplementary data). This approach was introduced by Stansly [41]. Any sample for which one of the test strains was significantly more resistant than the H37Rv strain was deprioritized.

Additional information for the biological dereplication was obtained from the evaluation of the spectrum of activity (SOA) against a microbial panel consisting of a Gram-positive bacterium—*Staphylococcus aureus*, Gram-negative bacterium—*Escherichia coli*, yeast—*Candida albicans*, and non-tuberculous mycobacterium—*M. smegmatis* (Supplementary data), all of which are widely used in HTS campaigns. Extracts with activity against one of the tested species have a higher chance of containing a known compound. Moreover, some antimicrobial compounds share similar structural cores or related targets or exhibit universal antimicrobial activity. Considering the long duration of TB treatment, it is preferable that new TB drugs possess a narrow SOA. This decreases the risk of bacterial resistance emerging in other microbial species during the long course of TB antimicrobial therapy.

The assessment of SOA reveals the specificity of the antimicrobial compounds according to the Gram reaction, taxonomy, and pathogenicity of the bacteria. Only extracts with low activity against yeasts and bacteria other than *M. smegmatis* were prioritized. Activity against *M. smegmatis* was not deprioritized due to the common molecular targets observed between *M. tb* and other mycobacteria. Therefore, activity against *M. tb* and other mycobacteria is commonly seen and does not diminish a compound’s potential use in TB treatment.

### Chemical dereplication by HPTLC-anti-*M. tb* bioautography-MS/NMR

To correlate biological dereplication with chemical identification/characterization of the hits [42], the HPTLC-anti-TB bioautography-MS/NMR approach was recently included into the drug discovery platform in the ITR laboratories [43]. The main goal of this technology is to facilitate prioritization of extracts in HTS campaigns for further processing and laying the groundwork for chemical dereplication at a very early stage of the isolation process. The bioautography assay [44] applies the avirulent, bioluminescent *M. tb* strain, mc^2^7000 luxABCDE [37, 45], which helps to overcome the challenges of working with a slow growing pathogen and the requirements of a BSL 3 laboratory. The anti-*M. tb* activity results of the samples could be determined after only 24 h, versus 8–10 days for common *M. tb* in vitro assays. The combination of TLC with MS and/or NMR allowed the chemical characterization of the separated compounds, and by using bioautography, their chemical profile could be matched with their biological activity.

### Reproducibility of anti-*M. tb* activity

All hits were divided into four priority groups based on their activity profiles. The first group contained hits with highly selective potency against replicating, non-replicating, and mono-resistant *M. tb* strains. The hits in the second group had (a) lower activity against replicating *M. tb*, (b) activity against at least one of the non-tuberculous species, and (c) in comparison to the first group, some toxicity against Vero cells. The hits in the third group had (d) even lower activity against replicating *M. tb*, (e) high activity against at least one non-tuberculous species, (f) toxicity against Vero cells, and (g) cross-resistance with mono-resistant *M. tb* strains. The fourth group contained the hits with the least favorable biological profile.

The source organisms of the prioritized hits of the four groups were refermented at medium scale, and extracted as before. The stock solutions of the newly fermented extracts were prepared at an accurate concentration in DMSO, and their activity was reassessed against replicating and non-replicating *M. tb*. All extracts that failed to reproduce anti-*M. tb* activity were removed from the hit prioritization list.

**Fig. 3:**
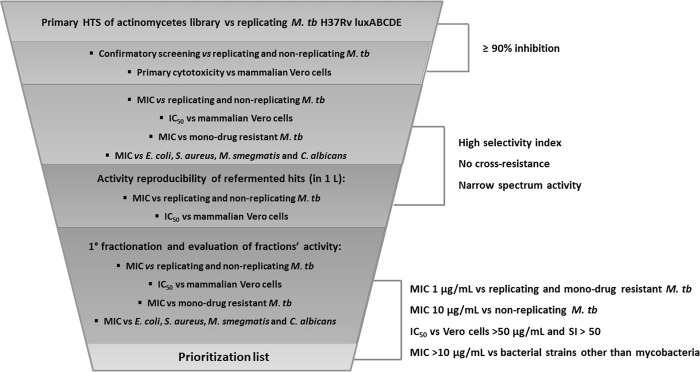
The prioritization workflow of the high-throughput screening campaigns of the actinomycetes libraries

The confirmed hits were fractionated by vacuum liquid chromatography (VLC) using reverse phase silica (C18) gel with a gradient elution 20–100% methanol in 20% increments and a final chloroform wash. The resulting six fractions were prepared in DMSO, and their activity was evaluated against replicating and non-replicating *M. tb*, mono-drug-resistant *M. tb* strains, *E. coli*, *C. albicans*, *M. smegmatis*, and ESKAPE (*Enterococcus faecium*, *S. aureus*, *Klebsiella pneumoniae*, *Acinetobacter baumannii*, *Pseudomonas aeruginosa*, and *Enterobacter aerogenes*) panels, as well as Vero cells (Supplementary data). Prioritization of the fractions was based on the same criteria as for the initial hits. The threshold MIC was defined as 1 µg/mL against replicating and mono-drug-resistant *M. tb*, 10 µg/mL for non-replicating *M. tb*, and >10 µg/mL against bacterial strains other than mycobacteria. The IC_50_ thresholds for Vero cells were 50 µg/mL, and SI values of >50 led to prioritization. All top priority fractions were selected for further studies (Fig. 3).

### Isolation and identification of active molecules from large scale fermentation cultures

After establishing the priority list of the strains, the source organisms of hits from the first group were refermented in larger volumes, e.g., 60 L. A bioactivity-guided fractionation (BGF) strategy was applied to isolate the anti-TB active compounds, i.e., all fractionation steps were done while monitoring IC_50_, MABA, and LORA values. The fractionation scheme included an initial separation via VLC (C18) to crudely separate the compounds of different polarities, followed by fractionation with a Sephadex LH-20 column to separate compounds of different polarity and molecular size. High-speed counter current chromatography (HSCCC) and (semi)preparative high-performance liquid chromatography (HPLC) were used in the final steps of purification.

The Bio-GUESS concept has been introduced recently to facilitate a more targeted isolation procedure of potential anti-*M. tb* leads using counter current separation (CCS) methods [46]. For this approach, the TLC-based “Generally Useful Estimate of Solvent Systems” (GUESS) strategy for the selection of a CCS solvent system [47] was combined with anti-TB bioautography to provide information about the optimal CCS solvent system for further chromatographic purification of the active compound(s). Avoiding individual, manual testing of many solvent system families, the application of the Bio-GUESS strategy offers two major advantages: (i) the solvent system prediction can be achieved without reference or target compounds, establishing a chemically untargeted and biologically fully targeted approach, (ii) CCS can be performed directly without the necessity of performing multiple partitioning experiments and in vitro assays. The selection of an HSCCC solvent system based on the Bio-GUESS methodology enables an isolation concept targeted to the biological activity, because the selection of the solvent system is based exclusively on the “sweet spot” of the active compounds [48].

The molecular weight of isolated compounds was determined via high-resolution mass spectrometry (HR-MS). Molecular formula prediction software was used to determine the elemental composition, and various public databases of mass spectral data were utilized for rapid dereplication. A more recent approach includes the use of MS/MS-based Molecular Networks algorithm which associated MS/MS spectra based on similar fragmentation patterns, as per the assumption that structurally related molecules will fragment in a similar way to give analogous patterns [49]. New molecules structurally related to known chemical scaffolds present in the database, can be rapidly assigned to specific structural families accelerating the characterization process.

Further structural elucidation was based primarily on NMR data collected with 400–900 MHz NMR instruments and including 1D ^1^H and various 2D (COSY, HSQC, HMBC, TOCSY, and ROESY/NOESY) experiments. The ^1^H NMR iterative Full Spin Analysis (HiFSA) was used for unambiguous assignment, confirmation of the proposed structure and validation of the newly identified natural product lead [50]. The method allows complete and accurate determination of all chemical shifts (*δ*^H^, in ppm), and ^1^H,^1^H spin–spin coupling constants (*J*_HH_, in Hz) present in a molecule from high-resolution ^1^H NMR spectra. It also enables resolution of highly complex ^1^H NMR signal patterns, yielding information about a molecular structure that otherwise is inaccessible and/or ignored.

### Hits from HTS campaigns

Table 1 provides detailed information about the number of samples selected in each step of the prioritization workflow in the seven major HTS campaigns performed on actinomycete extracts. As the net result of these efforts, 11 extracts were prioritized for extensive biological testing, isolation, and identification of the potentially new, anti-*M. tb* leads.

**Table 1:** Number of hits obtained in each step of HTS of actinomycetes extracts against *M. tb* over seven campaigns

Campaign	1	2	3	4	5	6	7
No. of screened extracts	65k	35k	20k	10k	20k	20k	10k
No. of primary hits	349	598	429	116	206	241	119
No. of confirmatory hits	92	42	50	18	15	25	24
No. of strains selected for small scale re-fermentation	22	15	16	5	2	2	3
No. of final prioritized hits	1	2	3	1	2	2	0

The first HTS campaign of 65k actinomycetes led to the prioritization of 22 extracts for further studies. Among them was a mycelial methanolic extract of *Nonomuraea sp*. MJM5123, the producing strain of the new macrocyclic tridecapeptide family, ecumicin and its analogs [33–35]. Ecumicin exerts selective bactericidal activity against *M. tb* in vitro, including non-replicating cultures, clinical isolates representing six major global clades (X001354 corresponding to the Indo-Oceanic lineage, X004439 and X004244 to the East Asian lineage, X005282 and X005319 to the Euro-American lineage, and X001354 to the East African Indian lineage), MDR and XDR strains. Ecumicin also shows in vivo activity; the complete inhibition of *M. tb* growth in mouse lungs was achieved after 12 doses at 20 or 32 mg/kg [33]. The absolute configuration and unequivocal structural confirmation of ecumicin was determined by NMR, X-ray and Marfey’s analysis. HiFSA analysis facilitated the elucidation of six ecumicin analogs [51]. Recently, the total synthesis of ecumicin provided an orthogonal structure proof as well as an alternative route to this compound, the in vitro activity of the synthetic molecule was also confirmed against *M. tb* [52].

The third HTS campaign of 20k actinomycete extracts led to the prioritization of three strains. The rapid dereplication of the ethyl acetate extract of *Streptomyces atratus*, MJM3502 revealed that the active components were rufomycins [36]. These compounds were originally isolated as ilamycins from several *Streptomyces* [53–56], and then re-isolated as rufomycins [57–59]. Ongoing studies are being performed in the ITR laboratories with rufomycin which was found to be highly active against *M. tb* and *M. abscessus*.

**Fig. 4:**
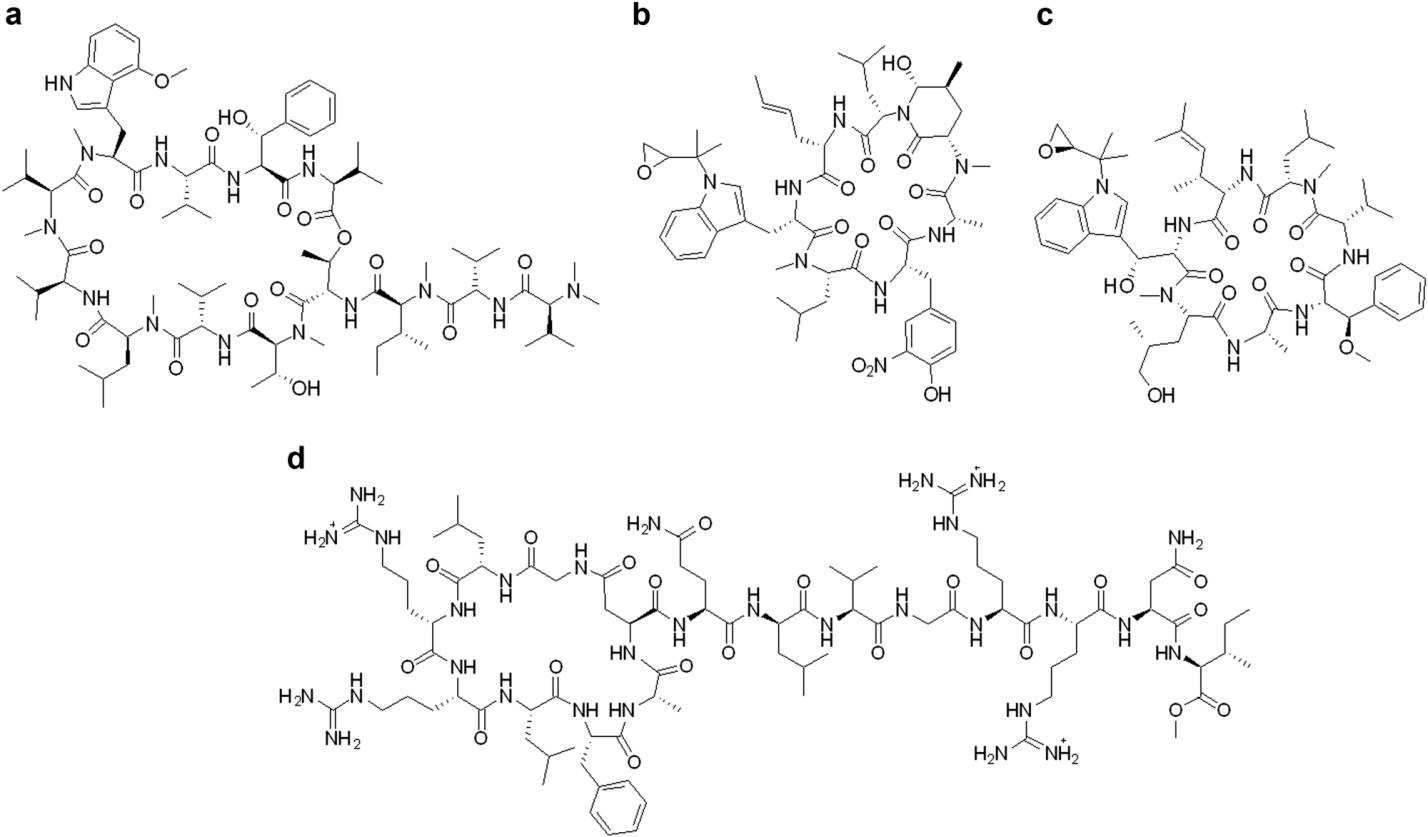
The chemical structures of **a** ecumicin, **b** rufomycin, **c** cyclomarin A, and **d** lassomycin

The lack of cross-resistance with existing anti-*M. tb* drugs of both ecumicin and rufomycin suggested a novel mechanism of action. The target has recently been identified as the ClpC1 ATPase a protein complex essential for the growth of *M. tb* [60, 61]. The recently discovered anti-*M. tb* natural products, lassomycin [62] and cyclomarin A [63, 64], have also been shown to share this target. Among them, ecumicin is currently the only lead with reported in vivo efficacy and, thus, the most advanced validated new anti-*M. tb* lead compound (Fig. 4).

## Conclusions

The selection of an adequate screening assay has always been an important part of drug discovery programs. Due to the slow growth and safety restrictions of *M. tb*, most of the screens were performed against the rapidly growing, *M. smegmatis* or BCG. However, the use of surrogate strains for screening risks missing compounds that are highly selective for *M. tb*. The experience with a screening and prioritization workflow, shared in this review, may facilitate the discovery of new active compounds with narrow spectrum activity and good safety profiles. So far, the phenotypic screening showed bigger potential than the target-based approach. All recently introduced anti-*M. tb* drugs or the promising candidates in clinical trials come from phenotypic screening. The new anti-*M. tb* drugs bring hope to more effective TB treatment.

## Supplementary information

Supplementary Data
