# Supplementary material for: Strategies in anti-Mycobacterium tuberculosis drug discovery based on phenotypic screening
Source: J Antibiot (Tokyo). 2019 Jul 11;72(10):719–28. doi: 10.1038/s41429-019-0205-9 (PMC6760628; doi:10.1038/s41429-019-0205-9)
Supplement: Supplementary file 1 — Supplementary Data [file 41429_2019_205_MOESM1_ESM.docx]

**SUPPLEMENTARY DATA**

**Construction of actinomycete culture extracts library**

One gram of each soil sample was suspended in 10 mL of 0.85% NaCl solution. Serial dilutions were spread on humic acid-vitamin agar (HV, humic acid 1 g, Na_2_HPO_4_ 0.5 g, KCl 0.71 g, MgSO_4_·7H_2_O 0.05 g, FeSO_4_·7H_2_O 0.01 g, CaCO_3_ 0.02 g, vitamin B solution 2 mL, agar 18 g, D.W. 1L, pH 7.0) and incubated at 28°C for 7 or 14 days. Vitamin B solution contained 0.5 mg each of thiamin-HCl (vitamin B1), riboflavin (vitamin G, vitamin B2), niacin (vitamin B3), pyridoxine-HCl (vitamin B6), inositol, Ca-pantothenate (vitamin B5), p-aminobenzoic acid (vitamin H1, vitamin Bx), and 0.25 mg of biotin (vitamin H, vitamin B7). Colonies that demonstrated morphological characteristics of actinomycetes were isolated by streaking on modified Bennett’s agar (BN, glucose 10 g, bacto peptone 2 g, yeast extract 1 g, beef extract 1 g, agar 15 g, D.W. 1L, pH 7.2) or Gauze’s No. 1 agar (GN1, soluble starch 20 g, KNO_3_ 1 g, NaCl 0.5 g, K_2_HPO_4_·7H_2_O 0.5 g, MgSO_4_·7H_2_O 0.5 g, FeSO_4_·7H_2_O 0.01 g, agar 18 g, D.W. 1 L, pH 7.2-7.4) and incubated at 28°C for seven days. Spores or aerial mycelium were collected by scraping the surface of each plate with sterile cotton wool soaked in 20% glycerol solution, the suspension was expressed into a sterile cryotube, and the glycerol stocks were stored at -80°C.

For activation of each strain, 0.1 mL of aliquots of the glycerol stock solutions were inoculated on Bennett’s agar plates and incubated at 28°C for 7 days. For the preparation of crude extracts, an agar block (1 cm x 1 cm) with abundant growth was inoculated into a 250 mL baffle flask containing 30 mL of one of three different liquid media, i.e., glucose-soybean starch (GSS, soybean meal 25 g, glucose 20 g, soluble starch 10 g, yeast extract 4 g, NaCl 2 g, CaCO_3_ 2 g, beef extract 1 g, K_2_HPO_4_ 0.25 g, D.W. 1L, pH 7.2), Bennett’s (BN, glucose 10 g, bacto peptone 2 g, yeast extract 1 g, beef extract 1 g, D.W. 1L, pH 7.2), and dextrin-yeast-corn steep liquor (DYC, dextrin 25 g, dry yeast 12 g, corn steep liquor 20 g, NaBr 1 g, CoCl_2_ 0.18 g, D.W. 1L, pH 7.0) media. After incubation at 28°C and 280 rpm for six days, cultures were centrifuged for 15 min at 15,000 g to separate the supernatant from the cell mass. The supernatant was then extracted with ethyl acetate (1:1, v/v) by vigorous vortex-mixing for 20 min. After the phase separation, the ethyl acetate layer was dried and re-dissolved in 3 mL methanol. The aqueous layer was heated at 60°C for 30 min to remove remaining ethyl acetate, freeze-dried, and re-dissolved in 3 mL water. The cell pellet was extracted with methanol (1:10, w/v) by vigorous vortex-mixing for 20 min and was then centrifuged for 5 min at 15,000 g to remove cellular debris. The methanol layer was dried and re-dissolved in 3 mL methanol. As a result, nine different extracts were generated for each actinomycete isolate (Figure 1). A 30 µL aliquot of each extract was distributed into 96-well plates, freeze-dried, and stored at 4°C. For screening purposes, each actinomycete extract was dissolved in 100 µL of dimethyl sulfoxide (DMSO), and the plate was left for 24 hours before screening to ensure all samples were sterilized.

**Primary HTS against *M. tb* H37Rv luxABCDE**

The primary HTS of the ECUM actinomycete library was performed using the luciferase expressing strain of *Mycobacterium tuberculosis (M. tb)*, H37Rv luxABCDE [37]. The actinomycete extracts and 7H12 medium (BD Difco, Franklin Lakes, NJ, USA) were added to a 384-well solid bottom white assay plate (Corning Inc., NY) using a liquid robotic handler Tecan Freedom EVO200 (Tecan Group Ltd, Männedorf, Switzerland). A volume of 50 μL of 7H12 medium was added to columns 1 and 24, whereas the columns 3 through 22 were filled with 50 μL of 7H12 medium and 1 μL of the actinomycete extracts. In the BSL3 laboratory, 1 μL inoculum of ~2×10^5^ CFU/mL *M. tb* H37Rv luxABCDE culture in 7H12 medium was added to all wells in columns 3 through 23 using a Microplate Replicator with 384 pins (Boekel Industries, PA). BD MGIT PANTA Antibiotic Mixture (polymyxin B, amphotericin B, nalidixic acid, trimethoprim and azlocillin) (BD, Franklin Lakes, NJ, USA) was added to 7H12 medium to suppress the growth of contaminants. The plates were incubated at 37°C for a period of seven days. The luminescence signal was measured using a Victor3 Plate Reader (PerkinElmer, MA).

**The confirmatory screening against non-replicating and replicating *M. tb***

All samples exhibiting inhibition ≥90% were followed up by screening against replicating and non-replicating *M. tb*. The assay plates were first filled with 7H12 medium; a volume of 100 μL was added to all wells in rows B through G in columns 1 and 12, and 98 μL to the columns 2 through 11. Next, 2 μL of the hit samples were added to all wells in rows B through G in columns 2 through 11.

For evaluation of the activity against actively growing *M. tb* in normoxic conditions, the assay was set up in 96-well clear bottom plates (Thermo Fisher Scientific, MA). The plates were inoculated with ~1×10^5^ CFU/mL *M. tb* H37Rv in a BSL3 laboratory and incubated at 37°C for a period of seven days. On day seven, 20 μL of 0.6 mM resazurin dye (Sigma-Aldrich, MO) and 12.5 μL of 20% Tween 80 (Sigma-Aldrich) were added to the assay plates, and fluorescence reduction was measured after an additional 24 h of incubation using a Victor3 Plate Reader.

For evaluation of the activity against non-replicating *M. tb* in hypoxic conditions, the assay was set up in 96-well white bottom plates (Thermo Fisher Scientific) which were inoculated with ~1×10^6^ CFU/mL of low oxygen-adapted *M. tb* H37Rv luxABCDE in the BSL3 laboratory. The plates were incubated under hypoxic conditions at 37°C for a period of 10 days, followed by 24-28 h of normoxic “recovery”. Then, the luminescence signal was recorded using a Victor3 Plate Reader. The actinomycete extracts exhibiting inhibition ≥90% in both assays were subjected to MIC determination against *M. tb*.

**MICs against *M. tb* and *M. tb* mono-drug-resistant strains**

The MICs against *M. tb* H37Rv (ATCC 27294) under normoxic replicating conditions were determined using the MABA as previously described [38]. The MIC value was defined as the lowest concentration effecting a reduction in fluorescence of 90% relative to that of controls.

The MICs against *M. tb* H37Rv under hypoxic, non-replicating conditions were determined using the Low Oxygen Recovery Assay (LORA) as previously described except that the luxABCDE reporter was used instead of the luxAB reporter gene [38]. The MIC was defined as the lowest concentration effecting a reduction in luminescence by 90%, relative to that of controls, after 10 days exposure to sample under hypoxic conditions followed by 28 hours of normoxic “recovery”.

The MABA was used to determine MICs against *M. tb* strains with mono-resistance to isoniazid (INH) (ATCC 35822), rifampin (RIF) (ATCC 35838), streptomycin (SM) (ATCC 35820), kanamycin (KM) (ATCC 35827), cycloserine (CS) (ATCC 35826), bedaquiline (TMC207) (ITM, Belgium), and the in house generated *M. tb* strains with mono-resistance to clofazimine (CLF), moxifloxacin (MOX), and capreomycin (CAP).

**MICs against non-tuberculous mycobacteria**

MICs for non-tuberculous mycobacteria were determined in a similar manner to that used for *M. tb*, except that *M. smegmatis* (ATCC 700084) and *M. abscessus* (ATCC 19977) were cultured in 7H9 medium (BD Difco, Franklin Lakes, NJ, USA) plus OADC supplement, at 37°C for 72 h prior to addition of 0.6 mM resazurin and 20% Tween 80. The fluorescence reduction was measured after an additional 4 h of incubation. *M. chelonae* (ATCC 35752) and *M. marinum* (ATCC 927) were cultured in 7H9 medium plus OADC supplement at 30°C for 72 h and 120 h, respectively. After addition of 0.6 mM resazurin and 20% Tween 80, the fluorescence reduction was measured after an additional 4 h of incubation. *M. avium* (ATCC 15769), *M. bovis* BCG and *M. kansasii* (ATCC 12478) were cultured in 7H9 medium plus OADC supplement at 37°C for six days (*M. avium*) and seven days (BCG and *M. kansasii*). Then, 0.6 mM resazurin and 20% Tween 80 were added, and fluorescence reduction was measured after an additional 24 h of incubation.

**MICs against non-mycobacteria**

*Enterococcus faecium* (ATCC 35667), *Klebsiella pneumoniae* (ATCC 13883), and *Enterobacter aerogenes* (ATCC 13048) were incubated in Mueller-Hinton II medium (Becton Dickinson, Sparks, MD, USA) at 37°C for 20 h. *Staphylococcus aureus* (ATCC 29213), *Acinetobacter baumannii* (ATCC BAA-747), and *Pseudomonas aeruginosa* (ATCC 27853) were incubated in Mueller-Hinton II medium at 37°C for 16 h. *Escherichia coli* (ATCC 25922) was cultured in Mueller-Hinton II at 37°C for 24 h. *Candida albicans* (ATCC 10231) was incubated in 1% Cellgro RPMI 1640 medium (Mediatech Inc., Manassas, VA, USA) supplemented with 1.8% D-(+)-glucose (ICN Biomedicals, Aurora, OH, USA) and 3.5% 3-(N-morpholino)propanesulfonic acid (MOPS) (Acros, NJ, USA) at 37°C for 48 h. Then, the optical density (OD) was measured at 570 nm, and the MIC was defined as the lowest concentration resulting in 90% reduction in absorption relative to that of untreated control cultures.

**Cytotoxicity in mammalian cells**

Vero cells (ATCC CRL-1586) were cultured in 10% Fetal Bovine Serum in Eagle’s Minimum Essential Medium supplemented with penicillin and streptomycin. The cells were prepared and washed in HBSS (1x pH = 7.4) and Trypsin-EDTA 0.25%, and their morphology was verified microscopically. The density was adjusted to 3-5×10^5^ cells/mL in MEM media, and 100 µL of the cell suspension was incubated with the samples at 37° C for 72 h. Then, 20 µL of 0.6 mM resazurin was added into each well and incubated for an additional 3-4 h. The fluorescence was determined by excitation/emission wavelengths of 530/590 nm. The primary cytotoxicity was determined as a reduction in fluorescence relative to untreated cells. The half-maximal inhibitory concentration (IC_50_) was defined as the concentration of sample effecting a reduction in fluorescence of 50% relative to untreated cells.

**Drug reference standards**

Isoniazid (INH), rifampin (RIF), moxifloxacin hydrochloride (MOX), pretomanid (PMD), linezolid (LIZ), clofazimine (CLF), kanamycin (KM), and metronidazole (MET) were purchased from Sigma-Aldrich Corp. (St. Louis, MO, USA). Bedaquiline (BDQ) was received from The Global Alliance for TB Drug Development.
